# Generational Differences in Fast Food Intake Among South-Asian Americans: Results From a Population-Based Survey

**DOI:** 10.5888/pcd11.140351

**Published:** 2014-12-04

**Authors:** Monideepa B. Becerra, Patti Herring, Helen Hopp Marshak, Jim E. Banta

**Affiliations:** Author Affiliations: Patti Herring, Helen Hopp Marshak, Jim E. Banta, School of Public Health, Loma Linda University, Loma Linda, California.

## Abstract

The goal of this study was to evaluate the association between generational status and fast food consumption among South-Asian Americans. We conducted a secondary analysis of data from the California Health Interview Survey for 2007, 2009, and 2011. After adjusting for control variables, South-Asian Americans of the third generation or more had a fast food intake rate per week 2.22 times greater than first generation South-Asian Americans. Public health practitioners must focus on ways to improve dietary outcomes among this fast-growing ethnic population in the United States.

## Objective

Poor dietary behaviors among South-Asian Americans (those from Bangladesh, Bhutan, India, Pakistan, Sri Lanka, Nepal, and the Maldives), increase with years of US residence and progressive generations (1,2), which in turn could explain the increased prevalence of cardiovascular disease among the population (3–6). To date, however, evaluation of generational differences in fast food intake among South-Asian Americans is lacking. Addressing fast food intake in adults in this population is critical, because such dietary practices have been significantly associated with increased risk of obesity and other chronic conditions (7). The goal of our population-based study was to evaluate the association between generational status and fast food consumption among South-Asian Americans.

## Methods

We used adult data files from the public-access California Health Interview Survey (CHIS) for 2007, 2009, and 2011. CHIS is a biennial population-based survey that uses a random-digit–dial sample, including both landline and cellular telephone numbers. Details on CHIS methods can be found elsewhere (8). For this study, 1,352 South-Asian Americans were included, representing an average annual estimate of 467,677 South Asians in California. CHIS defines South-Asian Americans as those from Bangladesh, India, Pakistan, and Sri Lanka. Fast food consumption was evaluated by using CHIS-reported number of times respondent consumed fast food in the past week. The CHIS-provided variable on country of birth for parents and self was used to create a generation variable: first generation (foreign born), second generation (born in United States with both parents foreign born), and third generation or more (born in United States with at least 1 parent born in the United States).

Control variables were age, sex, marital status (married, not married), educational status (Associate degree or less, Bachelor degree or more), body mass index (BMI) (kg weight/height m^2^), and poverty level (at or above 200% of the federal poverty level [FPL], below 200% FPL). Such variables were categorized on the basis of natural breakpoints in the population. In addition, we included English language proficiency to address the potential role of language in dietary practices to remain consistent with previous literature (2). The study was approved by Loma Linda University Institutional Review Board.

All analyses were survey-weighted by using the delete-1 jackknife method for variance and standard error estimation in Stata 12 (Stata Corp LP). To obtain tests for statistical significance in univariate analysis, negative binomial regression was performed with past-week fast food intake as the outcome variable. On checking for assumptions, an adjusted model for negative binomial regression was performed for past-week fast food intake. Design-based F statistics were used to assess significance of all analyses at *P* < .05.

## Results

The mean age of our study population was 38 years, and most (56%) were men (Table 1). Most of our population were married (67%), had a Bachelor degree or more (77%), were living at or above 200% FPL (83%), reported speaking only English or English very well or well (98%), and were first generation South-Asian Americans (83%). Mean BMI was 24.

**Table 1:** Characteristics of Study Population (n = 1,352), Generational Differences in Fast Food Intake Among South-Asian Americans^a^, California Health Interview Survey, 2007, 2009, and 2011^b^

Variable	Weighted % or Mean (95% Confidence Interval)
**Age, mean, y**	38.1 (37.2–39.1)
**Sex, %**	
Male	56.4 (52.4–60.3)
Female	43.6 (39.7–47.6)
**Marital status, %**	
Married	67.4 (62.8–71.7)
Not married	32.6 (28.3–37.2)
**Educational status, %**	
Bachelor degree or more	77.0 (73.6–80.3)
Associate degree or less	23.0 (19.7–26.4)
**Socioeconomic status, %**	
Living below 200% federal poverty level	16.9 (13.8–19.9)
Living at or above 200% federal poverty level	83.1 (80.1–86.2)
**English language proficiency, %**	
Speaks English only, very well, or well	97.7 (96.7–98.8)
Does not speak English well or does not speak English at all	2.3 (1.24–3.3)
**Generation level, %**	
First	83.2 (79.2–86.6)
Second	15.4 (12.2–19.3)
Third or more	1.3 (0.62–2.9)
**Body mass index (kg/m^2^** **)**, **mean**	24.1 (23.8–24.4)
**Survey year, %**	
2007	26.9 (24.3–29.5)
2009	34.4 (31.5–37.3)
2011	38.7 (35.9–41.5)

a The California Health Interview Survey defines South-Asian Americans as those from Bangladesh, India, Pakistan, and Sri Lanka.

b The total estimated California South-Asian American population from the 2007, 2009, and 2001 California Health Interview Survey was 467,677.

Several factors were significantly associated with past-week fast food intake among South-Asian American adults (Table 2). Increased fast food intake was associated with not being married compared with being married, (incidence rate ratio [IRR] = 2.09), having an Associate degree or less in comparison to having Bachelor degree or more (IRR = 1.44), and increasing generation (second generation, IRR = 2.07; third or more generation, IRR = 2.87) as compared with first generation. Conversely, yearly incremental increase in age was associated with a lower rate of past-week fast food intake (IRR = 0.97), as was living at or above 200% FPL, as compared with living below 200% FPL (IRR = 0.69).

**Table 2:** Unadjusted and Adjusted Negative Binomial Regression Analyses of Fast Food Intake Among South-Asian Americans^a^, California Health Interview Survey, 2007, 2009, 2011^b^

Variables	Unadjusted Model		Adjusted Model	
	IRR (95% CI)	*P* value	IRR (95% CI)	*P* value
**Generation level **				
First	1 [Reference]			
Second	2.07 (1.48–2.91)	<.001	1.32 (0.90–1.94)	.15
Third or more	2.87 (2.07–3.97)	<.001	2.22 (1.36–3.63)	.001
**Age**	0.97 (0.96–0.97)	<.001	0.98 (0.97–0.98)	<.001
**Sex**				
Male	1 [Reference]			
Female	0.92 (0.69–1.22)	.55	0.90 (0.70–1.15)	.39
**Marital status**				
Married	1 [Reference]			
Not married	2.09 (1.65–2.66)	<.001	1.30 (1.04–1.61)	.02
**Educational status**				
Bachelor degree or more	1 [Reference]			
Associate degree or less	1.44 (1.10–1.90)	.01	1.04 (0.75–1.45)	.81
**Socioeconomic status**				
Living below 200% FPL	1 [Reference]			
Living at or above 200% FPL	0.69 (0.53–0.91)	.01	0.90 (0.70–1.18)	.45
**English language proficiency**				
Speaks English only, very well, or well	1 [Reference]			
Does no speak English well or does not speak English at all	0.43 (0.18–1.05)	.07	0.63 (0.24–1.62)	.33
**Body mass index (kg/m^2^)**	0.98 (0.94–1.02)	.39	1.01 (0.99–1.04)	.35
**Survey Year**				
2007	1 [Reference]			
2009	1.06 (0.76–1.50)	.72	1.06 (0.80–1.40)	.70
2011	1.17 (0.90–1.51)	.24	1.13 (0.91–1.41)	.26

Abbreviation: IRR, incidence rate ratio; CI, confidence interval; FPL, federal poverty level.

a The California Health Interview Survey defines South-Asian Americans as those from Bangladesh, India, Pakistan, and Sri Lanka.

b The total estimated California South-Asian American population from the 2007, 2009, and 2001 California Health Interview Survey was 467,677.

Adjusted survey-weighted negative binomial regression analysis further demonstrated that third or more generation South-Asian Americans had a past-week fast food consumption rate 2.22 times higher than first generation South-Asian Americans. For every increasing year of age, an approximate 2% decrease in fast food consumption per week was noted. Similarly, not being married was associated with a 30% increased rate of past-week fast food consumption compared with those who reported being married.

## Discussion

Consistent with previous literature highlighting the negative effect of increased generational status on diet among minority populations (1,2,9), our study demonstrated that being South-Asian American of the third generation or more was significantly associated with an increased rate of past-week fast food intake. Such results show the need for targeted health promotion measures to improve dietary practices of such an ethnic group known to be at high risk for cardiovascular disease.

Increasing age was negatively associated with fast food intake, which is consistent with studies among other ethnic populations (10,11), further showing the need for health education measures addressing healthy diet for younger South-Asian American populations. Results further illustrated higher fast food consumption among those who were not married. This finding further corroborates an earlier study, where married respondents ate fast food on fewer occasions (11). The lack of association between English language proficiency and fast food consumption was expected, because the literature notes a high English proficiency in this population (12).

Our study has several limitations. The self-reported data in CHIS are susceptible to recall bias. Results are not generalizable to South-Asian American adults outside California, and small sample size further limited our ability to conduct sex-specific analysis. Despite such limitations, our study contributes to the limited body of literature on South-Asian Americans. Because this study was conducted using population-based survey data, findings are generalizable to South-Asian American residents of California. In addition, although most previous studies have used intake of foods such as fruits, vegetables, grains, and fat to assess diet, we used fast food intake, which does not require assessment of culturally relevant food items or energy adjustment of food types, which are often limitations of cross-sectional or public-use data sets. Additional studies providing insight into this association are necessary, in particular, questionnaires in South-Asian languages, longitudinal studies, and studies addressing generational differences in cardiovascular disease outcomes.
